# Discovery of Biomarkers for Osteosarcoma by Proteomics Approaches

**DOI:** 10.1155/2012/425636

**Published:** 2012-11-21

**Authors:** Yoshiyuki Suehara, Daisuke Kubota, Kazutaka Kikuta, Kazuo Kaneko, Akira Kawai, Tadashi Kondo

**Affiliations:** ^1^Department of Orthopedic Surgery, Juntendo University School of Medicine, 2-1-1 Hongo, Bunkyo-ku, Tokyo 113-8421, Japan; ^2^Division of Pharmacoproteomics, National Cancer Center Research Institute, 5-1-1 Tsukiji, Chuo-ku, Tokyo 104-0045, Japan; ^3^Department of Orthopedic Surgery, Tachikawa Memorial Hospital, 4-2-22 Nishikichou, Tachikawa, Tokyo 190-8531, Japan; ^4^Division of Musculoskeletal Oncology, National Cancer Center Hospital, 5-1-1 Tsukiji, Chuo-ku, Tokyo 104-0045, Japan

## Abstract

Osteosarcomas are the most common malignant bone tumors, and the identification of useful tumor biomarkers and target proteins is required to predict the clinical outcome of patients and therapeutic response as well as to develop novel therapeutic strategies. Global protein expression studies, namely, proteomic studies, can offer important clues to understanding the tumor biology that cannot be obtained by other approaches. These studies, such as two-dimensional gel electrophoresis and mass spectrometry, have provided protein expression profiles of osteosarcoma that can be used to develop novel diagnostic and therapeutic biomarkers, as well as to understand biology of tumor progression and malignancy. In this paper, a brief description of the methodology will be provided followed by a few examples of the recent proteomic studies that have generated new information regarding osteosarcomas.

## 1. Introduction 

 Osteosarcoma is the most common, nonhematopoietic, primary malignant bone tumor and most frequently occurs in the second decade, with 60% of patients under the age of 25 years [1]. After the initial diagnosis, patients usually receive multiagent preoperative chemotherapy and surgical resection of the tumor, followed by postoperative chemotherapy. Chemotherapy has improved the cure rate of patients with localized OS from 15%–20% achieved with surgery alone to approximately 70% [1, 2]. The response to preoperative chemotherapy is critical information for patients and the chemosensitive patients are divided into two groups based on the pathological features: the good responder (>90% tumor necrosis) and the poor responder (<90% tumor necrosis) [1, 2]. However, patients who have a poor response to chemotherapy often have a poor outcome and a high risk of developing metastasis compared to patients who have a good response to chemotherapy [1, 2]. Therefore, it is critical to identify proteins associated with chemoresistance as predictive biomarkers and novel theoretical targets in osteosarcomas. Additionally, despite significant progress regarding chemotherapy and improvements in the outcome for patients with localized osteosarcomas, patients who have metastases at diagnosis are not uncommon, and patients with metastases still have poor prognosis [1, 2]. Therefore, the development of a novel focus on the identification of prognostic indicators, and novel therapeutic targets that inhibit biological pathways known to contribute to osteosarcoma growth, are essential. 

 The use of high-throughput screening approaches, such as array-based comparative genomic hybridization analysis and cDNA microarray technology, allows for the screening of several thousand DNA and mRNA sequences and can identify the genes relevant to the diagnosis and clinical features of tumors [3–13]. These comprehensive studies have identified several genes that may be involved in the development or progression of osteosarcomas, and represent candidate biomarkers and/or drug targets [3–13]. However, in clinical applications, there are currently no specific markers available for predicting the prognosis and chemosensitivity of osteosarcomas. The identification of these factors could provide not only a new method for stratifying patients and selecting the treatment strategy, but could also provide novel therapeutic targets for osteosarcoma. Global protein expression studies, an approach known as “proteomics,” may also be more clinically relevant than genomic studies, since proteins directly regulate the aberrant tumor phenotypes. Moreover, DNA sequencing or measurement of mRNA expression cannot detect posttranslational modifications of proteins that affect their activity, such as phosphorylation, glycosylation, and acetylation, or differences in protein stability, and these factors play important roles in the malignant behavior of tumor cells [14–17]. Furthermore, many lines of evidence have indicated that there is discordance between the mRNA and protein expression [14–17]. Therefore, proteomic studies are becoming critical tools for understanding the biology of tumors, as well as for the identification of biomarkers for various cancers. In addition, the results obtained from proteomic studies are more easily applied to the clinical field, because of the potential use of specific antibodies.

 Recent advances in proteomic technology have made it possible to identify disease-related proteins in clinical samples, and extensive efforts are now being made to identify biomarkers of specific cancers that can be used for diagnostic or therapeutic purposes [15, 16, 18–25]. The standard proteomic techniques, such as two-dimensional gel electrophoresis (2DE) and mass spectrometry (MS), have been developing over the past three decades. From the end of the 1990s, through the development of high-throughput platforms, proteomics has become able to allow the simultaneous measurement of multiple protein products and protein modifications. These newlydeveloped technologies provide ways to detect crucial protein expression patterns corresponding to disease progression or responses to treatment and are now considered vital research tools [15, 16, 18–34]. Therefore, proteomic studies can be particularly useful to identify novel biomarkers and therapeutic targets of sarcomas. Our studies have identified some candidate proteins associated with a differential diagnosis [15, 16, 20, 21], prognosis [18, 19, 25], and in predicting the response to chemotherapy [16, 23] in bone and soft-tissue sarcomas. The following section describes how proteomic approaches have been applied to osteosarcoma and reviews many of the articles regarding proteomic studies of osteosarcoma.

 We identified approximately 30 papers about proteomic research on osteosarcoma during a search of PubMed (Table 1). In this paper, in order to discuss the contributions of proteomics to the identification of useful clinical biomarkers and targets for human osteosarcomas, we chose the articles that used actual patient samples, such as surgical materials and plasma, rather than information from human cell lines and animal studies (Table 1). In this paper, we briefly discuss the proteomic technologies, especially electrophoresis-based techniques, as well as the identification of biomarkers and novel targets for osteosarcoma identified by proteomic approaches. 

## 2. Proteomic Technologies and Analyses in Sarcomas

 Proteomics is the large-scale study of proteins, especially their structures and functions [35]. Technologies used in proteomics research include electrophoresis, mass spectrometric technologies, protein labeling, protein arrays, antibody-based approaches, imaging, and bioinformatics technology. As a result of the recent advances in these technologies, proteomics may provide powerful information for improved biomarker and novel therapeutic target discovery in malignant tumors. In particular, mass spectrometry technologies are now high throughput, allowing for the rapid and accurate identification of thousands of proteins present within a complex tumor specimen. Furthermore, a differential protein expression analysis can be used to compare tumors with normal tissues, and is able to identify a range of protein markers potentially related to the malignant features of tumors [35–37]. Bone and soft tissue sarcomas are relatively rare compared with other malignancies. The development of diagnostic and prognostic modalities, identification of novel therapeutic targets, and understanding of the mechanisms of sarcomagenesis are currently the main research priorities. Therefore, various strategies are now being employed to identify tumor-specific proteins in sarcoma using proteomics technologies.

Based on a search of the PubMed database, electrophoresis, specifically 2D difference gel electrophoresis (2D-DIGE) [15, 16, 19–21, 25, 32, 34, 38] and mass spectrometry, especially matrix-assisted laser desorption ionization mass spectrometry (MALDI MS) [39] and surface-enhanced laser desorption/ionization (SELDI) [26, 33, 40], have mainly been used to obtain protein expression profiles of bone and soft-tissue sarcomas. We briefly summarized both the advantages and disadvantages of main platforms for performing a proteomic analysis (Table 2). In this article, we mainly discuss 2D-DIGE, because this technology is the most commonly used method for obtaining protein expression profiles in proteomic studies of bone and soft tissue tumors [15, 16, 18–21, 25, 26, 32–34, 38–40].

### 2.1. 2D-DIGE

**Figure 1:**
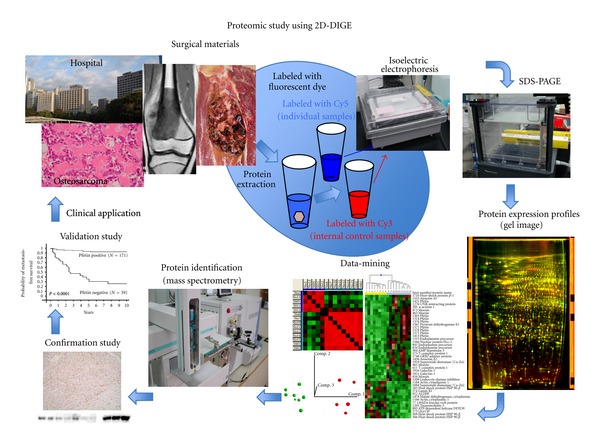
The process for 2D-DIGE-based target identification, confirmation, and validation. Surgical samples are collected from patients with osteosarcoma. Collected samples contain proteins associated with clinical information. All protein samples are labeled with different fluorescent dyes (The internal control sample is a mixture of a small portion of all individual samples labeled by Cy3, and the individual samples are labeled by Cy 5). The protein expression profiles are obtained using 2D-DIGE with highly sensitive fluorescent dyes. The protein expression profiles are analyzed to identify candidate biomarkers through data-mining using the proteomic profiles and clinical data. The protein expression levels are then confirmed by a western blot analysis and/or immunohistochemistry. The biomarker candidates are validated using additional large-variation cohorts to develop them for clinical applications.

 We routinely employ 2D-DIGE for biomarker identification [15, 16, 18–21, 25, 41, 42]. 2D-DIGE is an advanced variation of 2D-PAGE (two-dimensional polyacrylamide gel electrophoresis) and has the potential to solve many drawbacks of classical 2D-PAGE (Figure 1) [41, 42]. The standard 2D-PAGE technique employs isoelectric separation according to protein charge as a first dimension, coupled with MW resolution by polyacrylamide gel electrophoresis. Our 2D-DIGE method uses DIGE dyes which react with cysteine residues. There are only a few cysteines per protein and the cysteine-reactive dyes are highly soluble zwitterions. Therefore, these dyes are suitable for saturation labeling of all cysteine residues. As a result, the sensitivity for protein detection is improved and enables a successful 2D-PAGE analysis with of even low protein concentrations [41–43], and various studies have demonstrated the successful application of saturation labeling to detect protein differences in scarce samples derived from 1000 to 5000 protein spots. These two DIGE labeling options provide rapid methods for preparing differentially labeled samples for fluorescence-based proteome comparisons [41–43].

 Using our 2D-DIGE method, proteins were extracted from surgical specimens, and all protein samples were labeled with different fluorescent dyes before gel electrophoresis (Figure 1) [15, 16, 18–21, 25, 41, 42]. We first created a common internal control sample, which was a mixture of a small portion of all individual samples, and labeled it with a fluorescent dye that is different from the one used to label the individual samples. These differentially labeled internal control and individual samples are then mixed together and separated by both their pH and molecular weight ranges by 2D-PAGE (Figure 1). Laser scanning can be used to obtain the gel images, because all proteins are labeled with fluorescent dye before the gel electrophoresis (Figure 1). By normalizing the 2D image of each individual sample with that of the common internal control sample in the same gels, there is compensation for gel-to-gel variations, and reproducible results can be obtained across multiple gel images. These gel images provide data about protein spots as protein expression profiles. In the data analysis, the protein spots whose intensities are significantly different between the groups examined are identified using the Wilcoxon test, Hierarchical clustering, a principle component analysis, correlation matrix studies, and a support vector analysis using a data-mining software program (Figure 1). Proteins corresponding to the spots of interest are identified by mass spectrometry. Protein identification and differential expression are confirmed by a western-blotting analysis and/or immunohistochemistry using specific antibodies. Finally, in order to identify useful biomarkers and to develop clinical applications, we usually try to verify the value of biomarkers or targets using a large scale validation set which consists of clinical samples by an immunohistochemical analysis (Figure 1).

## 3. The Identification of Tumor-Specific Biomarkers and Therapeutic Targets in Osteosarcoma

 In osteosarcoma, the biology of malignant progression and tumorigenesis are still largely unknown. Therefore, protein and gene expression studies have mainly been used to provide a source of expression profiles and to obtain clues about the causes of osteosarcomas. The identification of tumor-specific biomarkers and therapeutic targets are the most important goals of global protein and gene expression studies. The current gene expression profiling technologies have been used to identify upregulated or downregulated genes associated with tumor progression, or that can be used to predict the malignant potential in osteosarcoma [3–6]. In this section, we mainly describe pertinent proteomic studies that have identified tumor-specific proteins corresponding to tumor progression and that can be used as prognostic biomarkers and therapeutic targets in osteosarcoma using patient materials. Two papers reported comparative proteomic studies using tissue samples; (i) osteosarcoma versus normal bone [32] and (ii) osteosarcoma samples versus benign tumors [34]. Additionally, one paper described a plasma proteomic study comparing osteosarcoma patients' plasma with plasma from patients with benign bone tumors (Table 1) [33]. 

 Folio et al. carried out comparative proteomic studies using five paired samples of osteosarcomas and normal bone to identify the proteins involved in malignant transformation and that were tumor-specific (Table 1) [32]. Additionally, the authors described that they could identify the proteins associated with metastasis and chemoresistance in their study. The five pairs of samples (tumor versus normal bone) were isolated form surgical tissue samples as cell lines. The study detected 56 differentially-expressed protein spots (*P* < 0.05) and 16 proteins were identified as having differences in their relative abundance in osteosarcomas and paired normal bones by nanoliquid chromatography/electrospray ionization tandem mass spectrometry (Nano-LC-ESI-MS/MS). Both alpha-crystallin B chain (*CRYAB*) and ezrin (*EZR*) were confirmed to be differentially expressed using an immunohistochemical analysis and real-time PCR. The confirmation studies revealed that tumor tissues had higher protein expression levels of both *CRYAB* and *EZR* than normal tissues. This study also demonstrated that there were significant differences in the metastasis and recurrence rates between positive and negative samples in terms of both *CRYAB* and *EZR.*

 Li et al. conducted a proteomic study of osteosarcoma to identify the specific protein markers of the disease and to improve the understanding of the tumorigenesis and progression of osteosarcoma (Table 1) [34]. Protein samples were extracted from five osteosarcoma tissue samples and five benign bone tumor samples (two osteoblastoma, two chondroblastoma, and one giant cell tumor). The protein expression profiles were acquired by comparing the osteosarcoma profiles with those of benign tumors using 2DE analyses. A total of 30 protein spots (*P* < 0.05) were detected as differentially expressed in this study, and 18 proteins were finally identified by mass spectrometry. These proteins included 12 upregulated proteins (VIM, TUBA1C, ZNF133, EZR, ACTG1, TF, etc.) and six downregulated proteins (ADCY1, ATP5B, TUBB, RCN3, ACTB, and YWHAZ). They confirmed the differences in the protein expression levels of TUBA1C and ZNF133 by the western-blotting and immunohistochemical analyses. The study concluded that these identified proteins might be potential biomarkers for osteosarcomagenesis and might represent novel therapeutic targets for the disease.

 Both studies (Folis et al. and Li et al.) identified osteosarcoma-specific proteins, as well as proteins associated with malignant progression and tumorigenesis in bone tumors, especially osteosarcomas [32, 33]. EZR was a common protein identified by both studies (Tables 3(a) and 3(b)). In a cDNA microarray study of osteosarcoma, EZR was also identified as a highly expressed gene in osteosarcomas, and that study suggested that EZR may have an important role in metastasis [5]. 

 EZR, an ezrin/radixin/moesin (ERM) family member, is evolutionarily conserved both structurally and functionally [44, 45]. By regulating membrane-cytoskeleton complexes, EZR has critical roles in normal cellular processes such as the maintenance of membrane dynamics, survival, adhesion, motility, cytokinesis, phagocytosis, and integration of membrane transport with signaling pathways [45, 46]. The activation of EZR is mediated by both exposure to PIP2 and phosphorylation of the C-terminal threonine (T567) [44, 47]. The deactivation of EZR is also important for physiologic functions, including the dynamics of actin-rich membrane projections. EZR has been implicated in various steps of the metastatic process, for example, as a conduit for signals between metastasis-associated cell surface molecules and signal transduction components [48]. 

 In osteosarcoma studies, EZR was necessary for metastasis, and a high expression of ezrin was associated with the early development of metastasis [49]. A relationship was also shown between high expression levels of EZR and a poor outcome in 19 pediatric osteosarcoma patients [49]. In addition, two articles that compared the EZR expression in high- and low-grade human osteosarcoma tumor samples demonstrated a clear correlation between ezrin expression and survival [50, 51]. Furthermore, some studies suggested that EZR phosphorylation is not only present in the early stage of metastasis, but also late in tumor progression, at the leading edge of large metastatsic lesions. More importantly, using dominant-negative mutants, antisense RNA or RNA interference, these experiments demonstrated that EZR overexpression is not only sufficient for metastatic progression, but it is also necessary in these experimental systems [49, 52]. Finally, the suppression of EZR protein expression significantly reduced the metastatic behavior in two murine models and it was also associated with a decreased Akt and mitogen-activated protein kinase (MAPK) activity [49, 52, 53]. These findings suggest that targeting EZR might improve the treatment of osteosarcoma, especially in metastasis cases. 

 Li et al. conducted a proteomic study to identify a plasma protein signature that was tumor-specific or that corresponded to the malignancy of osteosarcoma using SELDI-MS (Table 1) [33]. The study compared the plasma specimens between 29 patients with osteosarcomas and 20 with osteochondromas. They identified 19 ion peaks which had statistically significant differences in the protein expression levels between the osteosarcoma group and the benign bone tumor group (*P* < 0.001 and false discovery rate, 10%). The statistical analyses detected that there was a significant difference in the expression of one of the proteins (*m/z* 11 704) in the proteomic signature, which was found to be serum amyloid protein A by PMF. Serum amyloid protein A is highly expressed in the plasma samples of osteosarcoma patients. A Western blotting analysis also confirmed that there were high expression levels of serum amyloid protein A in the plasma of osteosarcoma patients compared to that of osteochondroma patients and normal subjects. That study concluded that the findings based on this plasma proteomic signature might be useful to differentiate malignant bone cancer from benign bone tumors, and also might contribute to early detection of a high-risk osteosarcoma group. 

 In summary, we believe that these proteins, which are tumor-specific and associated with the malignancy of osteosarcomas, may contribute to understanding the biology of the tumors and may be useful as biomarkers. An analysis of the functions of these proteins and their correlations with osteosarcoma would provide insight into the biology of osteosarcoma and improve the therapeutic management of osteosarcoma patients.

## 4. The Identification of Biomarkers of Chemosensitivity in Osteosarcoma

 The major prognostic factor in patients with localized osteosarcoma is the development of resistance to preoperative chemotherapy. Therefore, the identification of biomarkers of chemosensitivity in osteosarcoma is critical. Some gene expression studies have been conducted to identify genes associated with the chemosensitivity in osteosarcoma [3–6]. In this section, we mainly introduce pertinent proteomic studies that have previously identified markers of the response to chemotherapy in osteosarcoma. We found only two papers, which are our own studies, that have conducted protein expression studies of potential biomarkers of chemosensitivity in osteosarcoma using tissue samples (Table 1) [16, 23]. Another paper described a chemosensitivity study of osteosarcoma using patient plasma samples (Table 1) [26]. 

 In our study, we employed a proteomic approach to identify novel biomarkers of the chemosensitivity of osteosarcoma (Table 1) [23]. We used 12 biopsy samples, including six osteosarcoma samples from patients who were good responders and six osteosarcoma patients who were poor responders, according to the Huvos grading system. The protein expression profiles obtained by 2D-DIGE consisted of 2250 protein spots. We identified 55 protein spots (*P* < 0.01) whose intensity was significantly different between the two groups. Mass spectrometric protein identification demonstrated that these 55 spots corresponded to 38 distinct gene products, including peroxiredoxin 2 (PRDX2). The PRDX2 spots had higher intensity in the poor responder group, and we also confirmed the presence of an increased protein expression of PRDX2 in the poor responder group by a western blot analysis. In order to validate the prediction of chemosensitivity using the markers we identified, we performed a validation study using an additional four osteosarcoma samples, including two samples each from good responders and poor responders by a western blot analysis. The validation study demonstrated that the poor responders had higher PRDX2 expression levels compared to the good responders. We concluded that PRDX2 might be a candidate marker for chemosensitivity in osteosarcoma patients. In our other paper, by Kawai et al., we identified 10 protein spots which were found to be correlated with chemoresistance by 2D-DIGE, but the study did not identify the protein names associated with the spots, so we will not discuss the details of these findings here. 

 Li et al. conducted a plasma proteomic study to identify proteins that could be used to distinguish good from poor responders in osteosarcoma patients prior to the start of treatment (Table 1) [26]. Their study used two sets of 54 plasma samples that were collected from 27 prechemotherapy osteosarcoma patients and 27 postchemotherapy patients. The pre-chemotherapy samples included 14 good responders and 13 poor responders, and the postchemotherapy samples included 12 good responders and 15 poor responders. The analyses divided the subjects into two classes (consisting of good and poor responders) in both sets of plasma samples. In the post-treatment plasma set, 65 protein peaks were identified as the signature of the chemotherapeutic response. The levels of 29 protein peaks were higher and those of 36 protein peaks were lower in the plasma of poor responders. In the pretreatment plasma set, 56 protein peaks were identified, and the pre-treatment signature demonstrated that 32 and 24 protein peaks in the plasma of the poor responders were expressed at higher and lower levels, respectively, compared to good responders. These studies identified two plasma proteins, serum amyloid protein A and transthyretin, that appear to be especially sensitive markers. The expression of serum amyloid protein A was significantly higher in the plasma of the good responders, while transthyretin was more highly expressed in poor responders. These protein expression differences were confirmed by a western blot analysis. Both of these proteins are involved in innate immunity based on a protein database search. The authors concluded that the study might lead to the development of a simple blood test that can predict the response to chemotherapy in osteosarcoma patients and suggested that their findings might be corroborated by the notion that boosting the innate immunity in conjunction with chemotherapy leads to better anti-tumor activity, thus improving the overall survival of osteosarcoma patients. 

 Several reports have described that the histological response to preoperative chemotherapy has provided the most consistent and reliable prognostic indicator [1, 2, 5, 54]. The patients with localized disease whose tumors have undergone more than 90% necrosis have a 5-year survival of approximately 70%, while for those in whom the response falls short of 90%, survival rarely exceeds 40–50% [5, 54]. Furthermore, in comparison studies of osteosarcomas, the sample sets for chemosensitivity (poor responder versus good responder) were strongly correlated with the sets for prognosis (good outcome patients versus poor outcome patients). Therefore, we believe that the identified proteins associated with malignancy are also related to the chemosensitivity of the tumors. 

 Moreover, in a previous study, we have already discussed a comparison of the protein lists reported in Folio's and Li's studies (see above), with the Folio study examining tumor-specific and/or malignancy-related proteins (osteosarcoma versus normal tissue samples) and the Li study examining tumor malignancy (osteosarcoma versus benign bone tumor) (Table 3(a)). As noted above, we identified that EZR was a common protein identified in both studies (Table 3(b)). In this section, we also reviewed and organized the proteins lists from three papers: (i) Folio et al. reported tumor-specific and/or tumor malignancy-associated proteins (osteosarcoma versus normal tissue), (ii) Li et al. reported tumor malignancy-associated proteins (osteosarcoma versus benign bone tumors) and (iii) Kikuta et al. reported chemosensitivity- and/or tumor malignancy-related proteins (poor responder versus good responder) (Table 3(a)). During a further review of these studies, we found that not only EZR, but also the PRDX family were common proteins in the lists (Table 3(b)), with the study by Kikuta having identified PRDX2 and the Folio study having identified PRDX6 (Table 3(b)). 

 The PRDX family of proteins show peroxidase activities that degrade hydrogen peroxide (H_2_O_2_), as well as serving as alkyl hydroperoxides. In human tissues, six members of the family (PRDX1-6) have been identified so far [55]. Several papers have reported that PRDX proteins act as cytoprotective antioxidant enzymes, while PRDX expression was shown to enhance oxidative damage in some cells and tissues [55–60]. Recently, PRDX proteins have received increasing attention in the field of cancer biology. The analyses of cancer samples obtained from patients have revealed that there is increased expression of PRDX in malignancies of various organs and tissues [61–66]. In addition, the upregulation of PRDX proteins (specifically, 1, 2, 4, 5, and 6) may contribute to the resistance of tumors to chemotherapy and radiotherapy [67–73]. In addition, some papers have demonstrated in functional studies of PRDX proteins that they appear to influence the efficacy of cancer therapy not only by supporting the resistance of cancer cells, but also by promoting their invasiveness and metastasis [62, 74]. Therefore, the mechanism(s) underlying these functions of PRDX and information about their expression patterns may be essential for obtaining a better understanding of tumor biology and the development of new treatment strategies for osteosarcoma.

 In summary, based on the previous proteomic studies, there are several common proteins that have been identified as corresponding to chemoresistance and/or malignancy. Moreover, these proteins have been reported to be involved in malignancy and/or chemoresistance in other cancers [62, 67–74]. Therefore, these proteins should be major targets for development as diagnostic or prognostic biomarkers, and may also be useful for targeted therapy. 

## 5. Our Experiences with Proteomic Studies of Osteosarcoma

**Figure 2:**
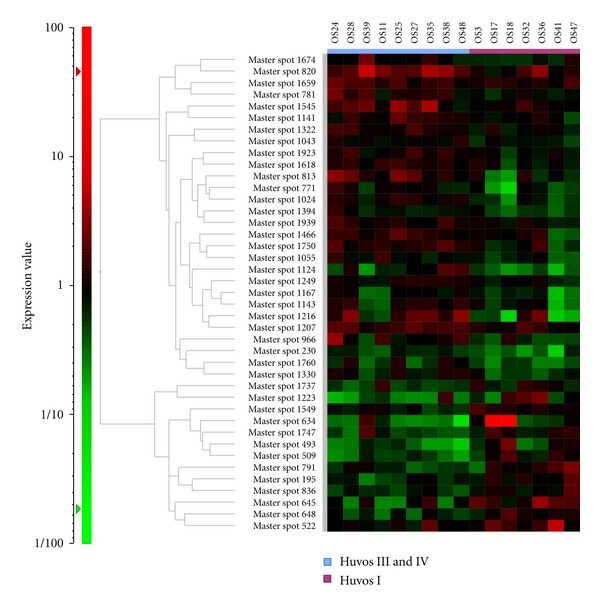
To identify proteins associated with the chemosensitivity of osteosarcoma, we conducted a 2D-DIGE study using osteosarcoma biopsy samples. A hierarchical cluster analysis of 16 osteosarcoma samples demonstrated that there were 41 protein spots that had different levels of intensity between samples. These 41 protein spots were identified from a total of 1465 protein spots (*P* < 0.05). The expected value of this study (from 1465 total protein spots and *P* < 0.05) was >73 protein spots. Therefore, the study design could not acquire a sufficient number of protein spots that had statistically significant differences in the expression levels between samples.

 Before the publication of Kikuta's paper, we conducted the 2D-DIGE analyses using 16 OS tissue samples to identify proteins corresponding to chemoresistance (Table 4). We compared nine osteosarcoma samples from good responders with seven osteosarcoma samples from poor responders. However, the sample set included osteosarcoma samples that were collected from elderly patients and from a trunk origin (pelvis), while typical osteosarcomas occur in young patients (<30 years) and in the extremities (Table 4). More importantly, the sample set was mixed, because several chemotherapy protocols, such as HD-MTX and HD-IFM, had been used (Table 4). Although this study identified 41 (of 1465) protein spots that were differentially expressed (*P* < 0.05), there were no statistically significant differences between the two groups of patients (good/poor responders) (Figure 2). On the other hand, Kikuta's study successfully identified 55 of 2250 protein spots (*P* < 0.01) which had statistically significant differences when the good responders and poor responders were compared. One of the keys to the success of the latter study was that Kituta's analyses used typical osteosarcoma samples, which were obtained only from tumors with an origin in an extremity and that were only obtained from young subjects. In addition, this study employed patient samples from only subjects who were treated with HD-IFM-based protocols. We think that the modified study design likely contributed to the successful identification of candidate proteins corresponding to chemosensitivity. 

 Oteosarcoma is a heterogeneous tumor, the etiology of which is still largely unknown. Three global gene expression studies using cDNA microarrays were conducted to determine genes that were differentially expressed with regard to the response to chemotherapy using osteosarcoma tissue samples. Ochi et al. identified a 60-gene signature predicting the treatment response on the basis of the 23000 cDNA microarray analyses and biopsy samples from 13 osteosarcoma patients. Mintz et al. reported a 104-gene signature associated with histologically evident responses to chemotherapy in osteosarcoma using an Affymetrix chip analysis of 30 pretreatment biopsy specimens. Man et al. described a 45-gene signature using the 9000 cDNA microarray analyses and 20 definitive surgical excision samples of osteosarcoma. However, these gene expression profiles lacked overlapping genes. Some reviews have suggested that the heterogeneity of osteosarcomas might be one of the reasons for the non-overlap [75]. 

**Figure 3:**
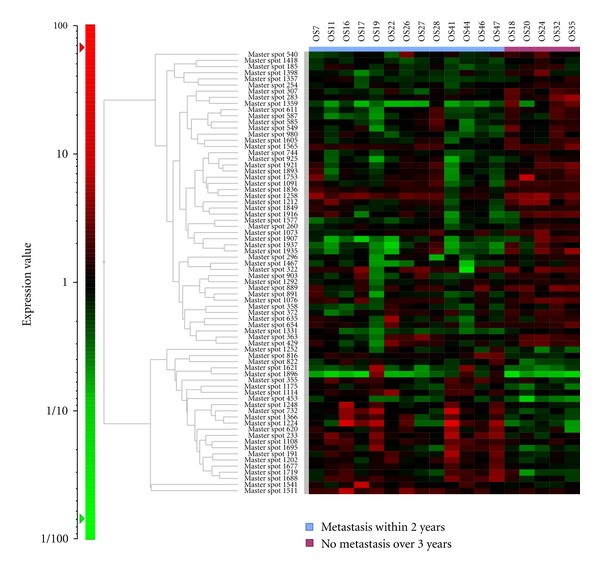
To identify proteins associated with the prognosis and malignant grade of osteosarcoma, we conducted a 2D-DIGE study using osteosarcoma biopsy samples. A hierarchical cluster analysis of 17 osteosarcoma showed that there were 72 protein spots that had different intensity out of a total of 1457 protein spots (*P* < 0.05). The expected value of this study (from 1457 total protein spots and *P* < 0.05) was >73 protein spots. Therefore, the study design also could not obtain a sufficient number of protein spots which had statistically significant differences.

 We conducted several proteomic studies using several kinds of surgical tissue samples and were able to identify candidate proteins associated with the prognosis of bone and soft tissue sarcomas, especially GISTs, Ewing sarcomas and synovial sarcomas [18, 19, 25]. Therefore, in our study that was not published, we conducted a proteomic approach to develop prognostic biomarkers for osteosarcoma using 2D-DIGE. In this trial, we used 17 surgical samples and divided the patients into two groups based on their prognosis ((i) the good prognosis group; five osteosarcoma patients who had not developed metastasis over a 3-year period and (ii) a poor prognosis group; 12 osteosarcoma patients who had developed metastasis within 2 years of the initial treatment) (Table 5). We compared the protein expression profiles between the two groups and identified that 72 of 1457 protein spots were differentially expressed (*P* < 0.05). However, these protein spots were not statistically different between the good and poor responder groups (Figure 3). As described above, we hypothesize that the heterogeneity of these osteosarcomas may have been one of the causes of the failure observed in this study. 

 The three types of tumors examined in our initial sarcoma studies; GISTs, Ewing sarcomas and synovial sarcomas, for which prognostic proteins could be identified in our studies, are associated with translocations or oncoproteins [18, 19, 25]. A general observation of both cDNA microarray- and proteomic-based expression profiling studies of sarcomas is that translocation-associated sarcomas are tightly clustered, whereas complex karyotype sarcomas tend to be less tightly clustered [15, 16, 75–79]. These findings and many reports indicate that translocation or oncoprotein-associated tumors are usually homogeneous tumors. On the other hand, complex karyotype sarcomas are usually heterogeneous tumors [15, 16, 75–79]. Therefore, based on our experiences with sarcoma proteomics, it likely to be more difficult to identify genes or proteins associated with the prognosis and chemosensitivity in heterogeneous tumors, like osteosarcoma, compared to homogenous tumors. Based on our experiences, we believe that it is vitally important to have a good study design which minimizes the noise by avoiding the inclusion of unsuitable samples. This will help to identify real and useful candidate proteins and will facilitate studies of highly heterogeneous tumors, such as osteosarcomas. 

## 6. Conclusion

 Several proteomic studies have identified candidate biomarkers relevant to a diagnosis of osteosarcoma, as well as for predicting its level of malignancy and chemosensitivity. 

We believe that these proteins are potentially useful biomarkers which may be useful for various clinical applications. However, such proteomic studies have not verified the value of biomarkers in a large validation set by immunohistochemistry or another reliable method. DNA sequencing or measurement of mRNA expression cannot predict the posttranslational modifications of proteins, but proteomic analyses are more directly linked to aberrant tumor phenotypes, and can more accurately reflect the status of tumors. However, compared with cDNA microarray analyses (50,000 probe sets), the sensitivity of the current 2D-DIGE analysis (5000 spots) is still unsatisfactory. In addition, whole-genome sequence tautologies have been developed. Because osteosarcomas are heterogeneous tumors, it is necessary to employ these technologies, such as CGH arrays, cDNA microarrays, whole-genome sequences and 2D-DIGE in combination, so that their individual disadvantages can be overcome, and to identify the most promising and useful biomarkers and molecular targets. These hybrid comprehensive studies consisting of genomics, transcriptomics, and proteomics experiments may provide important, novel clues for understanding the biology of tumors and for revealing biomarkers and therapeutic targets for osteosarcoma. 

## Figures and Tables

**Table 1:** Overview of protein expression studies using osteosarcoma samples published so far.

Sample type	Sample entity (cases)	Purpose of study	Method	Literature	Study contents
	Osteosarcoma (16)	Chemosensitivity	2D-DIGE	Kawai et al.	Chemosensitivity (poor responder (7) versus good responder (9))
Patient's tissue samples (osteosarcoma, normal bone, and benign tumor)	Osteosarcoma (5) and normal bone (5)	Tumor-specific and malignancy	2D-DIGE	Folio et al.	Tumor-specific proteins (osteosarcoma (5) versus normal bone (5))
	Osteosarcoma (12)	Chemosensitivity	2D-DIGE	Kikuta et al.	Chemosensitivity (poor responder (6) versus good responder (6))
	Osteosarcoma (5), chondroblastoma (2), osteoblastoma (2), and giant cell tumor (1)	Tumor-specific and malignancy	2DE	Li et al.	Tumor-specific proteins (osteosarcoma (5) versus chondroblastoma (2), osteoblastoma (2), and giant cell tumor (1))

Patient's plasma samples (osteosarcoma and benign tumor)	Osteosarcoma (29) and osteoblastoma (20)	Tumor-specific and malignancy	SELDI	Li et al.	Tumor-specific proteins (osteosarcoma (29) versus osteoblastoma (20))
	Osteosarcoma (54)	Chemosensitivity	SELDI	Li et al.	Tumor-specific proteins (poor responder (27) versus good responder (27))

	Osteosarcoma cell (1), osteoblastic cell (1)	Tumor-specific and malignancy	2DE	Spreafico et al.	Tumor-specific proteins (SaOS-2 (1) versus osteoblastic cell (1))
Cell line samples (osteosarcoma cell line versus normal bone or osteoblast cell)	Osteosarcoma cell (3), osteoblastic cell (1)	Tumor-specific and malignancy	2DE	Guo et al.	Tumor-specific proteins (U2OS (1), IOR/OS9 (1) and SaOS-2 (1) versus osteoblastic cell (1))
	Osteosarcoma cell (1), osteoblastic cell (1)	Tumor-specific and malignancy	2DE	Liu et al.	Tumor-specific proteins (SaOS-2 (1) versus osteoblastic cell (1))
	Osteosarcoma cell (1), osteoblastic cell (1)	Tumor-specific and malignancy	iTRAQ	Zhang et al.	Tumor-specific proteins of plasma membrane (MG63 (1) versus osteoblastic cell (1))
	Osteosarcoma cell (1), osteoblastic cell (1)	Tumor-specific and malignancy	2D-DIGE	Hua et al.	Tumor-specific proteins of plasma membrane (MG63 (1) versus osteoblastic cell (1))

Cell line sample (osetosarcoma cell)	Osteosarcoma cell (1)	Tumor specific	2D-DIGE	Niforou et al.	Characteristic proteins of U2OS cell line

Cell line sample (high metastatic osetosarcoma cell versus low metastatic osteosarcoma cell)	Osteosarcoma cell (2)	Metastasis	MALDI and 2D-DIGE	Cates et al.	Proteins corresponding to metastasis (high metastatic osetosarcoma cell (1) versus low metastatic osetosarcoma cell (2))

	Osteosarcoma cell (2)	Drug-sensitivity	2DE	Kang et al.	Drug-sensitivity: Ascochlorin (drug-treatment U2OS (1) versus no-treatment U2OS (1))
Cell line sample (drug treatment cell versus nontreatment cell)	Osteosarcoma cell (2)	Drug-sensitivity	2DE	Chang et al.	Drug-sensitivity: Ascochlorin (drug-treatment USOS (1) versus no-treatment USOS (1))
	Osteosarcoma cell (2)	Drug-sensitivity	2DE	Zhang et al.	Drug-sensitivity: DATS (drug-treatment SaOS2 (1) versus non-treatment SaOS2 (1))

Cell line sample (target inhibition or activation cell versus non-treatment cell)	Osteosarcoma cell (2)	Target related proteins	2D-DIGE	Li et al.	Target-related proteins: E2F1 (E2F1-activated SaOS2 (1) versus non-treatment SaOS2 (1))
	Osteosarcoma cell (4)	Target related proteins	2D-DIGE	Annunen- Rasia et al.	Target-related proteins: (i) OXPHOS, (ii) CI or CV, and (iii) mtDNA (inhibited SaOS2 (1) versus non-treatment SaOS2 (1))

2D-DIGE: 2-dimensional fluorescence difference gel electrophoresis, 2DE: 2-dimensional electrophoresis, MALDI: matrix-assisted laser desorption/ionization, and SELDI: surface-enhanced laser desorption/ionization.

**Table 2:** Summary of the platforms in proteomic analysis.

	Gel-based methods		Gel free methods		Microarray
	2D gel electrophoresis	2D-DIGE	SELDI and MALDI	LC-MS	Protein array
Advantage	Separation of large number of proteins	Reliable quantification Required small amount of proteins Reproducible Separation of large number of proteins	Automation Required small amount of proteins Possibility of quantification	Automation Required small amount of proteins Possibility of quantification	High-throughput Automation High reproducible Required small amount of proteins Sensitive detection for post translational modification

Disadvantage	Required large amount of proteins Nonautomation Limitation for large and small proteins in directions	Nonautomaition Limitation for large and small proteins in directions	Less reliable in protein direction Less detection of low abundance proteins	Less detection of low abundance proteins	Limitation for the total number of proteins

2D-DIGE: 2-dimensional fluorescence difference gel electrophoresis, SELDI: surface-enhanced laser desorption/ionization, MALDI: matrix-assisted laser desorption/ionization, LC-MS: liquid chromatography mass spectrometry.

**Table:** (a)

Protein name	Folio et al. (osteosarcoma versus normal bone)	Li et al. (osteosarcoma versus benign bone tumor)	Kikuta et al. (poor responder versus good responder)
40S ribosomal protein SA	Folio_4 (OS↓)		Kikuta_27 (poor↑)
Alpha-actinin-1			Kikuta_10 (poor↑)
Alpha-enolase			Kikuta_20, Kikuta_21 (poor↑)
Alpha1-antitrypsin		Li_11 (OS↑)	
Actin cytoplasmic 2	Folio_16 (OS↓)		
Actin-beta		Li_14 (OS↓)	
Actin-gamma1		Li_4 (OS↑)	
Adenylate cyclase 1		Li_13 (OS↓)	
Alpha crystallin beta chain	Folio_13 (OS↑)		
Alpha-enolase	Folio_8 (OS↓)		
Annexin A6			Kikuta_1 (poor↑)
ATP synthase mitochondrial F1 complex b polypeptide		Li_16 (OS↓)	
ATP synthase subunit b			Kikuta_39 (poor↓)
C-type lectin domain family 11 member A			Kikuta_4 (poor↑)
Carbonic anhydrase 1			Kikuta_52, Kikuta_54 (poor↓)
Chaperonin containing TCP1		Li_12 (OS↑)	
Clusterin precursor			Kikuta_2 (poor↑)
CNDP dipeptidase 2			Kikuta_17 (poor↑)
Coatomer protein complex		Li_3 (OS↑)	
Collagen alpha-1			Kikuta_55 (poor↓)
Desmoglein-1			Kikuta_46 (poor↓)
Elongation factor 1-gamma			Kikuta_34 (poor↓)
Eukaryotic initiation factor 4A-I			Kikuta_38 (poor↓)
Ezrin	Folio_7 (OS↑)	Li_9 (OS↑)	
Fascin	Folio_12 (OS↓)		
Ferritin light polypeptide		Li_7 (OS↑)	
Haptoglobin-related protein			Kikuta_31, Kikuta_53 (poor↓)
Heat shock 70 kDa protein 1			Kikuta_19 (poor↑)
Heat shock cognate 71 kDa protein	Folio_6 (OS↓)		Kikuta_23, Kikuta_25 (poor↑)
Heat shock protein beta 6	Folio_1 (OS↑)		
Heme binding protein 1	Folio_2 (OS↑)		
Hemoglobin subunit beta			Kikuta_7, 11, 15, 28, 36 (poor↑↓)
Keratin type II cytoskeletal 1			Kikuta_9 (poor↑)
Lamin B2		Li_1 (OS↑)	
Lamin-A/C			Kikuta_29 (poor↑)
LIM and SH3 domain protein 1	Folio_9 (OS↑)		
Lumican			Kikuta_43 (poor↓)
Myosin light chain 6 alkali smooth muscle and nonmuscle		Li_8 (OS↑)	
NADH-ubiquinone oxidoreductase			Kikuta_22 (poor↑)
Nucleophosmin	Folio_5 (OS↓)		
Peroxiredoxin 6	Folio_11 (OS↑)		
Peroxiredoxin 2			Kikuta_30 (poor↑)
PR65-A isoform			Kikuta_50 (poor↓)
Proteasome activator complex subunit 1			Kikuta_16 (poor↑)
Purine nucleoside phosphorylase			Kikuta_12 (poor↑)
Pyruvate kinase isozymes M1/M2	Folio_14 (OS↓)		
Reticulocalbin 3		Li_17 (OS↓)	Kikuta_13 (poor↑)
Ribose-phosphate pyrophosphokinase II			Kikuta_35 (poor↓)
Septin-11	Folio_15 (OS↑)		
Serum albumin			Kikuta_14, 41, 48 (poor↑↓)
Stress-70 protein			Kikuta_3, 24 (poor↑)
Thioredoxin reductase 1	Folio_10 (OS↑)		
Transferrin		Li_10 (OS↑)	
Trypsin-3 precursor			Kikuta_8, 26, 32 (poor↑↓)
Tubulin alpha-ubiquitous chain			Kikuta_45 (poor↓)
Tubulin beta-2A chain			Kikuta_47 (poor↓)
Tubulin beta-2C chain			Kikuta_37 (poor↓)
Tubulin beta-chain			Kikuta_44 (poor↓)
Tubulin-alpha-1C		Li_6 (OS↑)	
Tubulin-beta		Li_15 (OS↓)	
Tumor protein D54			Kikuta_40, 49 (poor↓)
Tyrosine 3-monooxygenase/tryptophan 5-monooxygenase activation protein		Li_18 (OS↓)	
Ubiquitin carboxyl terminal hydrolase isozyme L1	Folio_3 (OS↑)		
UV excision repair protein RAD23			Kikuta_33, 51 (poor↓)
Vesicle-fusing ATPase			Kikuta_42 (poor↓)
Vimentin		Li_2 (OS↑)	Kikuta_5, 6, 18 (poor↑)
Zinc finger protein 133		Li_5 (OS↑)	

**Table:** (b)

Protein name	Folio et al. (OS versus normal bone)	Li et al. (OS versus benign tumor)	Kikuta et al. (poor responder versus good responder)
40S ribosomal protein SA	Folio_4 (OS↓)	—	Kikuta_27 (poor↑)
Ezrin	Folio_7 (OS↑)	Li_9 (OS↑)	—
Heat shock cognate 71 kDa protein	Folio_6 (OS↓)	—	Kikuta_25 (poor↑)
Reticulocalbin 3	—	Li_17 (OS↓)	Kikuta_13 (poor↑)
Vimentin	—	Li_2 (OS↑)	Kikuta_5, _6, 18 (poor↑)
PRDX family	Folio_11 (PRDX6) (OS↑)	—	Kikuta_30 (PRDX2) (poor↑)

**Table 4:** Clinicopathological features of osetosarcoma tissue samples for chemosensitivity study.

Sample name	Age	Gender	Histological subtype	Site	Huvos grading	Preoperation chemotherapy agents	Mstastasis (months)	Followup (months)	Status
Huvos I									

OS03	9	Female	Telangiectatic	Proximal femur	1	MTX, ADR/CDDP	46	54	DOD
OS17	13	Female	Osteoblastic	Proximal tibia	1	HD-MTX	15	51	DOD
OS18	14	Male	Osteoblastic	Proximal tibia	1	HD-MTX, ADR/CDDP	—	73	NED
OS32	19	Male	Osteoblastic	Distal femur	1	HD-MTX, CDDP/ADR,	—	47	NED
OS36	63	Female	Osteoblastic	Metatarsus	1	IFO, CDDP/ADR	—	18	NED
OS41	18	Male	Osteoblastic	Pelvis	1	IFO, CDDP/ADR	At diagnosis	18	DOD
OS47	14	Male	Chondroblastic	Proximal femur	1	IFO, CDDP/ADR	At diagnosis	15	AWD

Huvos III and IV									

OS11	19	Male	Osteoblastic	Distal tibia	3	HD-MTX, ADR/CDDP	18	88	NED
OS25	13	Male	Li-Fraumeni	Distal femur	3	HD-MTX, ADR/CDDP	47	48	DOD
OS27	15	Female	Osteoblastic	Distal femur	3	HD-MTX, IFO, ADR/CDDP	At diagnosis	26	DOD
OS35	19	Female	Fibroblastic	Distal tibia	3	HD-MTX, CDDP/ADR	—	40	NED
OS38	18	Male	Osteoblastic	Distal tibia	3	IFO, CDDP/ADR	—	26	NED
OS48	8	Male	Osteoblastic	Proximal humerus	3	IFO, CDDP/ADR	—	14	NED
OS24	14	Female	Osteoblastic	Distal femur	4	HD-MTX, CDDP/ADR,	—	60	NED
OS28	9	Female	Osteoblastic	Proximal tibia	4	HD-MTX, CDDP/ADR,	6	51	NED
OS39	16	Male	Chondroblastic	Proximal tibia	4	IFO, CDDP/ADR	—	26	NED

**Table 5:** Clinicopathological features of osetosarcoma tissue samples for prognosis study.

Sample name	Age	Gender	Histological subtype	Site	Huvos grading	Preoperation chemotheapy agents	Mstastasis (months)	Followup (months)	Status
Metastasis within 2 years									

OS07	43	Male	Osteoblastic	Pelvis	2	MTX, ADR/CDDP	6	27	DOD
OS11	19	Male	Osteoblastic	Distal tibia	3	HD-MTX, ADR/CDDP	18	88	NED
OS16	67	Female	Osteoblastic	Distal femur	—	—	19	38	DOD
OS17	13	Female	Osteoblastic	Proximal tibia	1	HD-MTX	15	51	DOD
OS22	61	Male	Osteoblastic	Spine	—	—	5	7	DOD
OS26	19	Male	Osteoblastic	Distal femur	1	HD-MTX, CDDP	19	45	AWD
OS27	15	Female	Osteoblastic	Distal femur	3	HD-MTX, IFO, ADR/CDDP	At diagnosis	26	DOD
OS28	9	Female	Osteoblastic	Proximal tibia	4	HD-MTX, CDDP/ADR,	6	51	NED
OS41	18	Male	Osteoblastic	Pelvis	1	IFO, CDDP/ADR	At diagnosis	18	DOD
OS44	18	Male	Osteoblastic	Distal femur	1	IFO/VP	At diagnosis	3	DOD
OS46	18	Male	Chondroblastic	Distal femur	2	IFO, CDDP/ADR	At diagnosis	17	NED
OS47	14	Male	Chondroblastic	Proximal femur	1	IFO, CDDP/ADR	At diagnosis	15	AWD

No metastasis over 3 years									

OS18	14	Male	Osteoblastic	Proximal tibia	1	HD-MTX, ADR/CDDP	—	73	NED
OS20	12	Male	Osteoblastic	Distal femur	1	HD-MTX, ADR/CDDP, IFO	—	68	NED
OS24	14	Female	Osteoblastic	Distal femur	4	HD-MTX, CDDP/ADR,	—	60	NED
OS32	19	Male	Osteoblastic	Distal femur	1	HD-MTX, CDDP/ADR,	—	47	NED
OS35	19	Female	Fibroblastic	Proximal tibia	3	HD-MTX, CDDP/ADR	—	40	NED
